# High Glucose-Induced Alterations in Regucalcin Expression in Podocytes and Their Potential Consequences

**DOI:** 10.3390/ijms27031602

**Published:** 2026-02-06

**Authors:** Olga Żołnierkiewicz, Dorota Rogacka

**Affiliations:** Laboratory of Molecular and Cellular Nephrology, Mossakowski Medical Research Institute, Polish Academy of Sciences, 80-308 Gdansk, Poland; drogacka@imdik.pan.pl

**Keywords:** regucalcin, SERCA, ER stress, podocytes, T2DM, DKD

## Abstract

Regucalcin (RGN) is a multifunctional regulator of intracellular calcium signaling, implicated in cellular homeostasis and stress responses. While aberrant RGN activity has been associated with diabetic kidney disease (DKD), existing studies have primarily focused on its role in proximal tubular cells. Whether RGN is expressed in podocytes and how its expression responds to diabetic-like stimuli remain largely unexplored. Podocyte injury under diabetic conditions is a critical event in DKD pathogenesis. Therefore, in this study, we aimed to investigate whether podocytes express RGN and how its expression is affected under high-glucose (HG) conditions. To address these questions, we employed quantitative real-time PCR, Western blotting, fluorescence-based protein staining, and immunohistochemical analysis of renal sections. Our results confirmed RGN expression in podocytes and revealed its dysregulation at both the mRNA and protein levels under HG conditions. Additionally, we identified the subcellular localization of RGN and a significant association with sarco/endoplasmic reticulum calcium ATPase (SERCA), a key enzyme regulating endoplasmic reticulum (ER) calcium storage and the ER stress response. Altered RGN expression in podocytes exposed to HG concentrations may contribute to the progression of DKD, possibly through the disruption of intracellular calcium homeostasis.

## 1. Introduction

Extracellular calcium ion (Ca^2+^) concentrations are approximately 10,000-fold higher than intracellular levels. This significant gradient requires tightly regulated mechanisms to maintain Ca^2+^ homeostasis and ensure the fidelity of Ca^2+^-dependent signaling. Among these regulatory mechanisms is regucalcin (RGN), a highly conserved 33.388 kDa [1] Ca^2+^-binding protein whose expression declines with age (also known as senescence marker protein-30; SMP30) [2]. RGN plays an important role in intracellular Ca^2+^ homeostasis by acting as a suppressor of Ca^2+^ signaling. Unlike classical Ca^2+^-binding proteins, RGN lacks a canonical EF-hand motif [3], highlighting its unique structural and functional properties. First identified in the late 1970s, RGN is predominantly expressed in the liver [4] and renal cortex [5] (particularly in tubular cells). Renal RGN plays multifaceted roles in cellular Ca^2+^ regulation, apoptosis and redox balance.

RGN promotes renal Ca^2+^ reabsorption. Lin et al. demonstrated that androgens increase RGN expression in male rats, reducing urinary Ca^2+^ loss. Orchiectomy reduced RGN and increased urinary Ca^2+^, reversible by testosterone supplementation, suggesting that RGN supports Ca^2+^ retention in renal tubules [6].

RGN exerts anti-apoptotic effects in renal epithelial cells. According to Yamaguchi, RGN overexpression inhibits apoptosis induced by Tumor Necrosis Factor alpha (TNF-α) and radiation by preventing DNA fragmentation, caspase-3 activation, cytochrome c release and expression of pro-apoptotic proteins like Bcl-2-associated X protein (Bax) and Apoptotic Protease Activating Factor 1 (Apaf-1), while increasing anti-apoptotic Bcl-2 levels [7].

In renal cells, RGN also plays an antioxidant role. Marques et al. showed RGN reduces reactive oxygen species (ROS) and regulates enzymes like nitric oxide synthase and mitochondrial dehydrogenases [8]. As these enzymes are essential for mitochondrial energy metabolism and redox homeostasis, their regulation by RGN suggests that the protein functions as a critical modulator of intracellular oxidative stress responses. Supporting this notion, Okada et al. presented that RGN-knockout mice exhibited increased oxidative stress markers in tubular cells, and lower expression has been linked to age-related chronic kidney diseases [9].

RGN may serve as an important modulator of sarco/endoplasmic reticulum Ca^2+^-ATPase (SERCA) activity [10,11], thereby contributing to intracellular Ca^2+^ homeostasis by limiting excessive Ca^2+^-dependent signaling. SERCA is a membrane-bound pump that actively transports Ca^2+^ from the cytosol into the endoplasmic reticulum (ER), the primary intracellular calcium reservoir. Proper SERCA function is essential for replenishing ER calcium stores and maintaining cellular Ca^2+^ balance. Disruption of this mechanism can lead to ER Ca^2+^ depletion, triggering ER stress and activation of pro-apoptotic signaling pathways.

RGN activity is altered in metabolic diseases, such as type 2 diabetes mellitus (T2DM) [12], a chronic condition characterized by insulin resistance and hyperglycemia, which frequently progresses to chronic kidney disease, with diabetic kidney disease (DKD) being one of its major complications [13]. A hallmark of DKD is glomerular filtration barrier (GFB) disruption, ultimately with the leakage of plasma proteins into the urine. The GFB is a highly specialized tri-layer structure composed of (i) endothelial cells, (ii) the glomerular basement membrane and (iii) podocytes, terminally differentiated epithelial cells with a complex morphology. As a critical component of this barrier, podocytes exhibit a cell body, primary processes, and interdigitating foot processes that enwrap the glomerular capillaries. These foot processes form the slit diaphragm, a highly dynamic structure crucial for selective ultrafiltration of GFB. Maintenance of slit diaphragm architecture, and therefore GFB integrity, is critically dependent on the podocyte actin cytoskeleton. This cytoskeletal scaffold not only provides mechanical resilience but also facilitates rapid reorganization in response to hemodynamic [14] or metabolic signals [15].

Podocyte cytoskeletal remodeling and detachment represent a pivotal pathogenic event in the development of DKD [16,17]. Ca^2+^ is a critical regulator of podocyte actin cytoskeletal organization. Ca^2+^-dependent remodeling of the actin cytoskeleton is mediated through the modulation of Rho GTPase signaling pathways [18]. These molecular switches control actin polymerization and cytoskeletal organization, and are essential for maintaining podocyte shape, adhesion, and motility. The essential role of Ca^2+^ signaling in podocyte homeostasis is further underscored by transcriptomic analyses, which have identified Ca^2+^-responsive gene networks involving platelet-derived growth factor-BB (PDGF-BB; a key regulator of cell proliferation and migration), a component of the mTORC2 complex involved in cytoskeletal dynamics (RICTOR), and MIR17HG (a long non-coding RNA with emerging roles in podocyte signaling) [19]. Moreover, Djenoune et al. showed that Ca^2+^ signaling is crucial for foot process development in zebrafish [20].

Although the presence of RGN in podocytes has not been empirically demonstrated to date, its known functions in Ca^2+^ handling, cell signaling and physiology raise the intriguing possibility that it may participate in modulating podocyte condition. Considering the central role of Ca^2+^ in maintaining podocyte architecture and function, we hypothesize that RGN might represent an unrecognized regulator of glomerular homeostasis. In this study, we sought to determine whether podocytes are capable of expressing RGN and to explore how its expression is modulated under high-glucose (HG) conditions, mimicking in vivo diabetic milieu. These findings may shed new light on the potential consequences of RGN expression dysregulation in podocytes within the context of DKD.

## 2. Results

**Figure 1:**
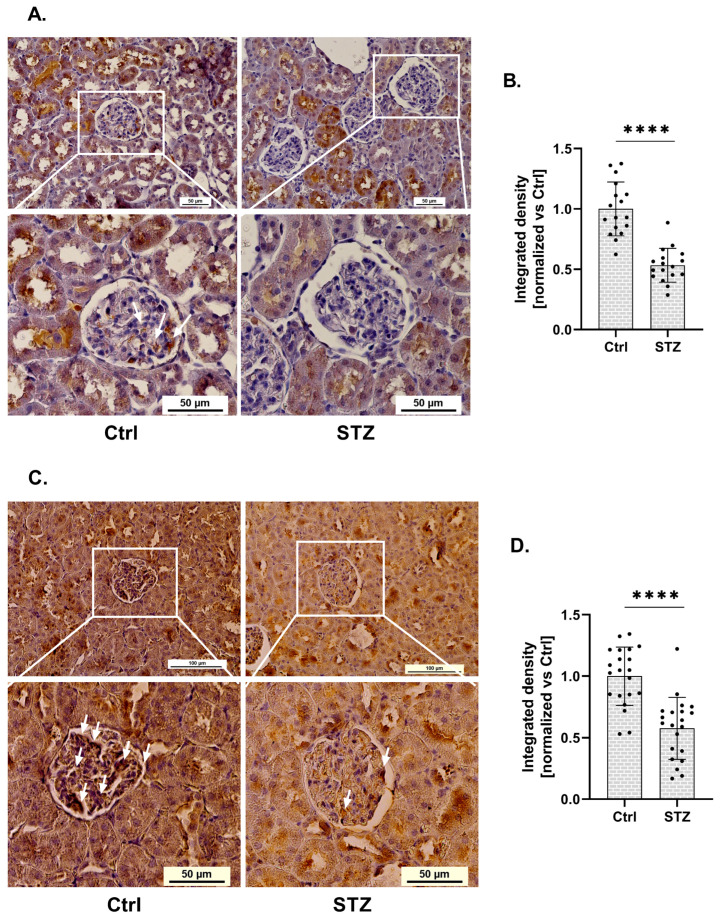
Expression of RGN and SERCA in glomeruli of diabetic rats. (**A**). Immunohistochemical analysis of RGN in diabetic and control (Ctrl) rat glomeruli. Scale bar shown for the full image (**up**) (50 µm) and for the close-up view of the glomeruli (**down**) (50 µm). Images were acquired with a 20× objective. Arrows indicate areas of positive RGN staining in renal glomeruli. (**B**). Reduction in RGN expression in glomeruli of diabetic rats. Data are shown as mean ± SEM, **** *p* < 0.0001, *n* = 17, one biological replicate, Student’s *t*-test. (**C**). Representative immunohistochemical staining of SERCA in glomeruli from Ctrl and diabetic rat kidneys. Scale bars: 100 µm (overview, **top panels**) and 50 µm (magnified glomerular region, **bottom panels**). Images were captured using a 20× objective lens. Arrows indicate areas of positive SERCA staining in renal glomeruli. (**D**). Quantitative analysis of a marked decrease in glomerular SERCA expression in diabetic rats. Results are presented as mean  ±  SEM; **** p < 0.0001, *n* = 21, one biological replicate, Student’s *t*-test.

Using immunohistochemistry, we demonstrated statistical changes in RGN protein expression within the glomeruli of kidneys isolated from streptozotocin (STZ)-induced diabetic rats (Figure 1A). RGN expression in glomeruli isolated from diabetic rats was reduced by 47%, compared to control (Ctrl) conditions (Figure 1B). This downregulation was statistically significant and localized specifically to the glomerular compartment.

Given the established involvement of RGN as a SERCA activity modulator, we next investigated whether changes in RGN expression were associated with alterations in SERCA expression. Immunohistochemical analysis revealed a significant alteration in SERCA protein levels within the glomeruli of kidneys collected from (STZ)-induced diabetic rats (Figure 1C). Quantification showed a 42% decrease in glomerular SERCA expression in diabetic animals relative to non-diabetic controls (Figure 1D). This reduction was statistically significant and confined specifically to the glomerular region.

**Figure 2:**
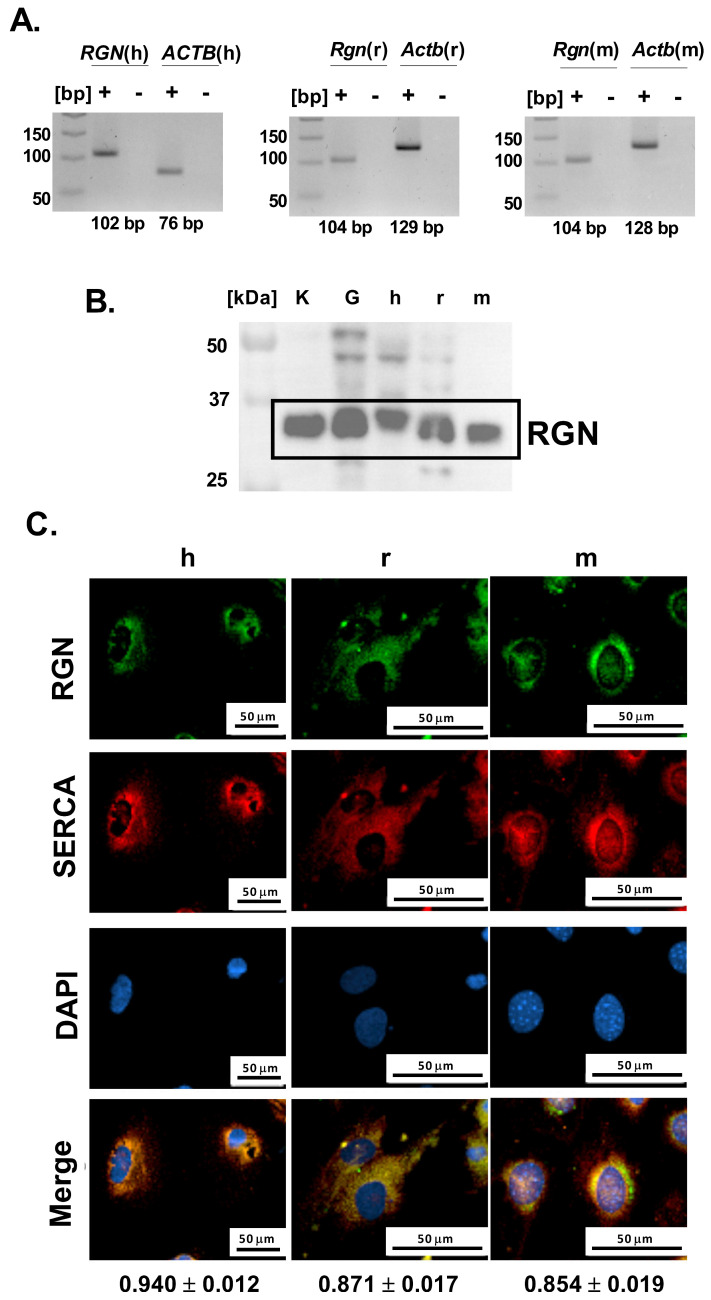
Identification of RGN in podocytes. (**A**). *Rgn* gene amplification. The primer pairs were used to amplify human *RGN* (*RGN*(h)), human *ACTB* (*ACTB*(h)), rat *Rgn* (*Rgn*(r)), rat *Actb* (*Actb*(r)), mouse *Rgn* (*Rgn*(m)), mouse *Actb* (*Actb*(m)). “+” indicates amplification reactions performed in the presence of cDNA template, whereas “-“ corresponds to no-template control reactions. (**B**). RGN protein identification in human (h), mouse (m) and primary rat (r) podocytes. Kidney (K) and glomerular (G) tissue lysates were included as positive controls to validate antibody specificity and confirm the expected molecular weight of RGN (**C**). Cellular localization of RGN and correlation with SERCA. h—immortalized human podocytes; r—primary rat podocytes; m—immortalized mouse podocytes. The results are reported as Pearson correlation coefficients. Data are shown as mean  ±  SEM, *n* = 12 cells. Scale bar = 50 µm; 40× objective magnification.

Further, to investigate whether podocytes contribute to glomerular RGN expression, we next examined their endogenous potential for RGN production at both the mRNA and protein levels. Specific amplification products corresponding to *Rgn* mRNA were successfully obtained from both immortalized human and mouse podocyte cell lines and primary podocyte cultures (Figure 2A). The lack of detectable bands in the no-template reactions confirms the specificity of the amplification and excludes nonspecific products such as primer–dimer formation. The amplicon sizes were consistent with the predicted lengths of 102 bp for human and 104 bp for both rat and mouse podocyte *Rgn* transcripts. Amplification products were visualized alongside parallel reactions for *Actb* as an internal control, confirming *Rgn’s* integrity and reaction specificity. These data establish that podocytes from all three species express *Rgn*, confirming the gene’s active transcription in these cells.

Consistent with transcript detection, immunoblotting confirmed the presence of RGN protein in all examined podocyte cell types (Figure 2B). Immunoreactive bands were detected near the 37 kDa molecular weight marker band, aligning with the expected molecular mass of 34 kDa as reported by the antibody manufacturer. Positive control rat samples, including kidney and isolated glomeruli showed comparable banding patterns, supporting the antibody’s specificity and validating the detection method. These results demonstrate that podocytes not only transcribe the RGN gene but also translate it into a detectable protein product.

To characterize the cellular distribution of RGN in podocytes, we examined its intracellular localization using immunofluorescence microscopy. Across both immortalized human and mouse podocyte cell lines and from primary podocyte cultures, RGN was consistently detected within the cytoplasm, exhibiting a pronounced perinuclear accumulation pattern (Figure 2C). This spatial distribution suggests a cytoplasmic role for RGN, with potential enrichment near the endoplasmic reticulum (ER) or other perinuclear organelles involved in Ca^2+^ handling.

Simultaneously, microscopic imaging showed a strong Pearson correlation between RGN and SERCA in human (0.94 ± 0.01), rat (0.87 ± 0.02), and mouse (0.85 ± 0.02) podocytes, suggesting the colocalization of RGN and SERCA (Figure 2C). The observed colocalization proposes a close spatial and potentially functional interaction between RGN and SERCA, implicating RGN in the regulation of Ca^2+^ transport within the ER of podocytes. Such a role would be consistent with RGN’s previously described function as a modulator of intracellular Ca^2+^ pumps and suggests a link to podocyte homeostasis.

**Figure 3:**
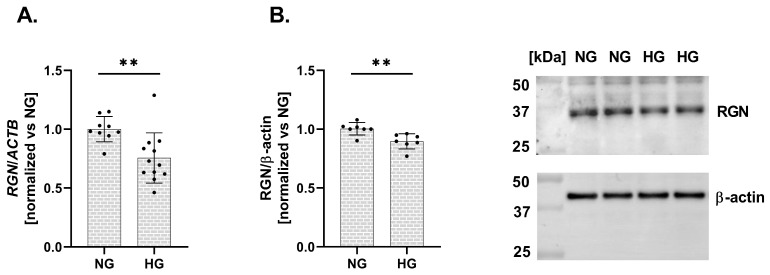
Alterations in RGN expression in a human podocyte cell line under HG conditions. *RGN* mRNA (**A**) and protein (**B**) expression levels are downregulated under HG conditions compared to NG. Data are shown as mean  ±  SEM, ** *p* = 0.0054, *n* = 3 biological replicates, each performed in triplicate or more, Student’s *t*-test (for mRNA expression), and ** *p* = 0.0053, *n* = 2 biological replicates, performed in triplicate or quadruplicate, Student’s *t*-test (for protein expression).

Finally, we assessed the impact of HG conditions on RGN expression in a human podocyte cell line. Exposure to elevated glucose levels resulted in a significant downregulation of RGN expression at both the mRNA and protein levels. The expression level of mRNA *RGN* exhibited a 24% decrease (vs. NG; Figure 3A). Similarly, RGN protein level presented a 11% reduction (vs. NG; Figure 3B). A reduction in RGN expression in response to HG suggests that podocytes become more susceptible to Ca^2+^ dysregulation and structural injury, potentially contributing to the pathogenesis of DKD.

## 3. Discussion

Our renal tissue analysis revealed changes in RGN expression within the glomeruli of diabetic rats. The results indicate a marked downregulation of RGN protein. This finding is consistent with the data reported by Hsu et al., who performed a mass spectrometry-based proteomic analysis of glomeruli from diabetic rats and identified broad alterations in protein expression profiles [21]. The alterations in RGN expression observed in our study are consistent with previous reports suggesting that diabetes-induced changes in glomerular proteins, including RGN, may reflect early pathological events in DKD [21]. Although decreased renal RGN expression has been associated with reduced RGN levels in urinary exosomes from diabetic rats and patients [12], further investigation is required to clarify its utility as a biomarker.

The detection of protein within glomerular structures substantiates the assumption that podocytes are capable of expressing RGN. This hypothesis is further corroborated by the detection of RGN expression at both transcript and protein levels, providing robust molecular evidence of its presence in podocytes. Notably, RGN expression was consistently identified across three distinct podocyte models. To our knowledge, this is the first study to demonstrate RGN expression in podocytes, thereby offering novel insights into the molecular landscape of these cells.

RGN exhibited a primarily cytoplasmic distribution, with prominent accumulation in the perinuclear area of podocytes. Tsurusaki et al. observed translocation of exogenous RGN to nuclei isolated from liver cells [22]. The nuclear translocation of RGN was established as an early event and it was not related to a classical nuclear localization signal, which typically governs active transport into the nucleus. Instead, its nuclear accumulation may reflect passive diffusion mechanisms or association with cytoplasmic structures proximal to the nuclear envelope, potentially facilitating localized Ca^2+^ buffering or signaling regulation at the nuclear–cytoplasmic interface. Interestingly, RGN translocation to the nucleus was correlated with inhibition of nuclear protein kinase and protein tyrosine phosphatase activities [22], playing crucial roles in regulating nuclear processes involved in cell cycle progression, DNA replication, transcription, and RNA processing [23]. Furthermore, Yamaguchi et al. have demonstrated that RGN inhibits the activity of Ca^2+^-dependent DNA endonucleases, thereby reducing nuclear DNA fragmentation [24]. RGN’s nuclear translocation suggests (i) its involvement in transcriptional regulation, indicating importance in cellular nuclear signaling, and (ii) protective function, where tightly regulated Ca^2+^ signaling is essential for maintaining genomic stability and cell viability. The perinuclear localization of RGN in podocytes may also suggest potential regulatory roles of protein in transcriptional control in these cells.

In our study, we demonstrated that RGN expression is significantly downregulated in podocytes under HG conditions, both at the transcript and protein levels. However, the reduction in protein abundance was notably less pronounced than that observed for mRNA, suggesting that RGN degradation may be attenuated in a diabetic context. This discrepancy implies the involvement of mechanisms that prolong RGN protein stability and maintain its levels despite reduced transcription. Two mechanisms appear particularly relevant: (i) stabilizing post-translational modifications (PTMs) and (ii) spatial sequestration limiting proteolytic access. PTMs such as acetylation and glycosylation are known to reduce protein turnover by masking ubiquitination sites or shielding proteolytic cleavage regions. Under hyperglycemic conditions, non-enzymatic glycation and the formation of advanced glycation end-products (AGEs) can induce covalent cross-links that render proteins more resistant to degradation [25,26]. Moreover, increased O-GlcNAcylation has been shown to compete with ubiquitination, thereby stabilizing proteins through interference with proteasomal targeting [27]. It is therefore plausible that under HG conditions, RGN can undergo both enzymatic and non-enzymatic PTMs that collectively extend its half-life and buffer against transcript-level downregulation. A second mechanism likely contributing to RGN stabilization involves its subcellular localization. RGN has been shown to associate with intracellular membranes, including the ER. The ER forms specialized contact sites with mitochondria, known as mitochondria-associated membranes (MAMs) [28], which create spatially restricted microdomains [29] within the cytoplasm. Proteins localized to such domains can be physically shielded from cytosolic degradation machinery. Indeed, mitochondria have been proposed to act as “holdout compartments” where proteins are protected from ubiquitin ligases and proteasomes [30,31]. In this context, RGN’s localization may limit its exposure to proteolytic systems, thereby prolonging its intracellular stability. Collectively, these observations point to potential mechanisms that may underlie the relative preservation of RGN protein levels under diabetic conditions. However, further experimental studies will be necessary to elucidate the precise molecular determinants governing RGN stability in this context.

The present study was conducted using a male in vivo model to ensure consistency with established diabetic protocols and to limit biological variability associated with fluctuating hormone levels. However, emerging evidence indicates that renal expression of RGN is subject to sex-specific hormonal regulation. In particular, testosterone has been shown to upregulate RGN expression [6], whereas estradiol exerts a downregulating effect [32]. These observations suggest that biological sex and hormonal milieu may modulate RGN levels in both physiological and disease contexts. While the current findings offer new potential insights into podocyte dynamics under hyperglycemic conditions, further studies, including those involving both sexes and incorporating hormonal modulation, are warranted to delineate the full spectrum of RGN regulation and enhance translational relevance.

To place our findings into a broader biological context, it is important to consider potential mechanisms that may underlie RGN downregulation in podocytes. Diabetes promotes excessive oxidative stress through hyperglycemia-induced ROS generation. Experimental animal models demonstrated RGN suppression under oxidative stress [33,34]. Supporting these observations, Junk et al. reported that RGN transcription is sensitive to the cellular redox state, with regulation mediated through promoter binding sites, suggesting that the extracellular signal-regulated kinase (ERK) signaling contributes to the observed protein downregulation [35]. Our previous work showed that HG exposure induces oxidative stress in podocytes, accompanied by increased NADPH oxidase activity and ROS generation [36]. Furthermore, Piwkowska et al. demonstrated HG increases albumin permeability across podocyte monolayers through NOX4-mediated oxidative stress [37]. Taken together, these findings suggest that the reduction in RGN expression in podocytes could represent an additional mechanism through which hyperglycemia-induced oxidative stress promotes increased permeability of the GFB; however, this requires further investigation.

The consequences of HG-induced downregulation of RGN expression in podocytes are most plausibly linked to altered Ca^2+^ handling. This downregulation of RGN may disrupt intracellular Ca^2+^ homeostasis in podocytes, potentially compromising Ca^2+^-dependent signaling pathways and contributing to cellular dysfunction under hyperglycemic conditions. Physiological cellular Ca^2+^ homeostasis is maintained by the interplay of plasma membrane transport systems, including Store-Operated Ca^2+^ Entry (SOCE), transient receptor potential channels (TRPCs), and Ca^2+^ adenosine triphosphatases (ATPases), facilitating Ca^2+^ influx and efflux. Additionally, intracellular compartments, such as the ER, utilize SERCA, ryanodine receptor (RyR), and inositol 1,4,5-trisphosphate receptor (IP_3_R) channels to regulate Ca^2+^ storage and release. Szrejder et al. found a significant upregulation of TRPC6 protein expression in podocytes and rat glomeruli under hyperglycemic conditions [38], suggesting enhanced Ca^2+^ influx into the cytosol. TRPC6 functions as a critical facilitator of Ca^2+^ influx in podocytes, thereby influencing cellular signaling pathways that are implicated in the pathogenesis of DKD. Tao et al. have implicated that SOCE initiated by receptor activation at the plasma membrane contributes to podocyte injury under hyperglycemic conditions. Increased Ca^2+^ influx through SOCE channels has been associated with reduced expression of nephrin, a critical component of the slit diaphragm, indicating structural compromise [39]. Finally, the literature underscores an association between disruptions of SERCA activity in renal tissue and the pathophysiology of T2DM. SERCA is essential for the active transport of Ca^2+^ from the cytosol into the ER lumen [40].

The significant reduction in SERCA expression observed in parallel with decreased RGN levels in glomeruli, along with their colocalization in podocytes, suggests a functional interplay between these two proteins. This spatial association implies that RGN may serve as a modulator of SERCA activity, contributing to the regulation of intracellular Ca^2+^ homeostasis by attenuating Ca^2+^-dependent signaling. In light of the available literature, we hypothesize that the colocalization of SERCA with RGN represents a physiological state in podocytes and that perturbations of this interaction may trigger metabolic alterations contributing to podocyte dysfunction. Alterations in RGN expression under HG conditions may influence Ca^2+^ equilibrium by modulating SERCA function, potentially leading to ER Ca^2+^ depletion. Such disturbances highlight a putative role of RGN in safeguarding podocyte integrity. Guo et al. have reported a significant reduction in SERCA expression and activity in the kidney in db/db mice (an animal model of T2DM) [41]. These findings were corroborated by analyses of the kidney cortex from STZ-induced diabetic mice and an immortalized mouse podocyte cell line under HG conditions, in which the researchers observed a significant reduction in the expression and activity of SERCA. This SERCA dysfunction led to the compromise of Ca^2+^ storage in the ER, thereby inducing ER stress and triggering ER stress-mediated apoptotic pathways. Impairments in SERCA expression resulted in ER Ca^2+^ depletion, causing the accumulation of misfolded proteins that activated ER stress-related apoptotic responses through C/EBP homologous protein (CHOP) and Jun N-terminal kinase (JNK) and potentially cell death mechanisms in podocytes under diabetic conditions [42]. RGN has been shown to enhance the transcription of SERCA. Lai et al.’s study demonstrated that RGN overexpression is associated with increased expression of SERCA at both the mRNA and protein levels, suggesting a potential regulatory interaction of RGN-ER and enhancing the ability of the ER to sequester Ca^2+^ and attenuating cytosolic Ca^2+^ elevations in response to external stimuli [10]. Additionally, Yamaguchi et al. have presented the ability of RGN to enhance SERCA activity, which was entirely suppressed when digitonin (a compound that disrupts membrane lipids) or N-ethylmaleimide (an agent that modifies sulfhydryl groups; -SH) was introduced. These observations indicate that RGN likely associates with membrane lipids near SERCA, interacts with -SH groups and promotes Ca^2+^-dependent phosphorylation [11]. Despite these findings, the precise mechanisms that govern the RGN-dependent regulation of SERCA expression and activity remain to be fully elucidated.

**Figure 4:**
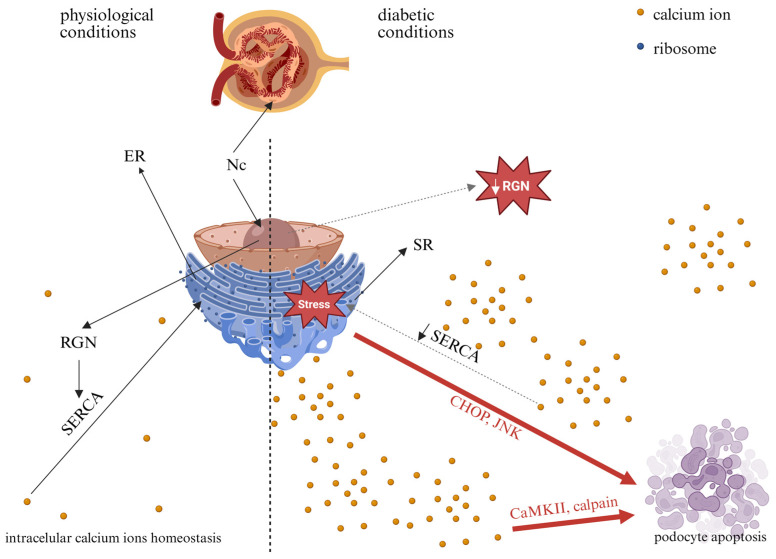
Potential consequences of high-glucose-induced alterations in RGN expression in podocytes. Nc—nucleus; ER—endoplasmic reticulum; SR—sarco/endoplasmic reticulum; RGN—regucalcin; SERCA—sarco/endoplasmic reticulum Ca^2+^ adenosine triphosphatase; CHOP—C/EBP homologous protein; JNK—c-Jun N-terminal kinase; CaMKII—Ca^2+^/calmodulin-dependent protein kinase. Solid black arrows indicate physiological processes, dashed black arrows indicate potential changes in diabetes, and red arrows indicate pathways that may lead to apoptosis. Created in BioRender. Kulesza, T. (2026) https://BioRender.com/jyx5yz3, accessed on 7 January 2026.

Collectively, in the diabetic milieu, RGN-related dysfunction could contribute to podocyte injury and increased GFB permeability, a hallmark of DKD. Figure 4 illustrates the potential consequences of altered RGN expression in podocytes under HG conditions. Further studies are needed to delineate the mechanistic interplay between RGN expression, Ca^2+^ homeostasis, and podocyte pathology in the context of hyperglycemia. A better understanding of these molecular mechanisms could potentially inform future hypothesis-driven approaches to targeted therapeutic strategies in DKD.

## 4. Materials and Methods

### 4.1. Approval by the Ethics Committee

All experimental procedures were performed in accordance with directive 2010/63/EU and approved by the Local Bioethics Committee in Bydgoszcz (no. 28/2025). Wistar rats used in this study were supplied by the internal animal facility of the Mossakowski Medical Research Institute, Polish Academy of Sciences (Warsaw, Poland).

### 4.2. Experimental Animals and Metabolic Cage Procedures

In accordance with a previously established experimental protocol [43], 10-week-old male Wistar rats were utilized in the experiment. In brief, the experiment involved healthy and STZ-induced diabetic Wistar rats (six per group) kept under a controlled 12 h light–dark cycle with unrestricted access to chow and water, where diabetes was confirmed by fasting glucose levels exceeding 250 mg/dL on the third day after induction. Metabolic cage studies were conducted from day 15 to day 17 following a 24 h adaptation period, during which urine output, water intake, and urinary albumin concentration were measured, and the animals were subsequently euthanized for kidney collection. In our previous studies, we demonstrated that in this model of diabetes, isolated glomeruli from STZ-treated rats exhibit impaired filtration function [44]. Metabolic parameters in healthy and diabetic Wistar rats are summarized in Table 1.

### 4.3. Isolation and Maintenance of Rat Podocytes

Female Wistar rats weighing between 100 and 120 g were used as the source of podocytes. The isolation of podocytes was conducted using a previously reported method [45]. As a concise description, the kidneys were dissected, and glomeruli were isolated using sequential sieving into RPMI 1640 (Sartorius AG, Göttingen, Germany) supplemented with 10% fetal bovine serum (FBS) and 1% penicillin–streptomycin (complete medium). Glomeruli were cultured in collagen-coated flasks for 5–7 days, after which podocytes were separated, reseeded, and grown under standard conditions. Experiments were conducted on podocytes maintained in culture for 12–20 days.

### 4.4. Human and Mouse Podocyte Cell Line Culture

An immortalized human podocyte cell line (generously provided by Prof. Moin A. Saleem, University of Bristol, United Kingdom) was cultured following an established protocol. For the maintenance of proliferative activity, podocytes were initially grown in RPMI 1640 complete medium (Sartorius AG) at a permissive temperature of 33 °C. Upon reaching approximately 80% confluence, differentiation was initiated by shifting the incubation temperature to 37 °C. Cells were subsequently maintained under these conditions for 10–14 days to enable the development of a fully differentiated podocyte phenotype. Experimental procedures on human podocytes were carried out within an incubation period ranging from day 12 to day 20. To evaluate the impact of HG conditions, differentiated human podocytes were exposed to RPMI 1640 complete medium with 30 mM D-glucose for up to 5 days, according to our previous studies [37]. These experimental conditions were compared to normal glucose conditions, in which cells were incubated for 5 days in RPMI 1640 complete medium containing 5.6 mM D-glucose (NG).

Mouse SVI (catalog no. 400495, CLS Cell Lines Service GmbH, Eppelheim, Germany) podocytes were cultured according to the manufacturer’s protocol with Dulbecco’s Modified Eagle Medium (5.6 mM glucose) that was supplemented with 5% fetal bovine serum.

### 4.5. Extraction of Total RNA and Quantitative Real-Time PCR Assessment

Total RNA was extracted from cell lysates using the RNeasy Mini Kit (Qiagen N.V., Venlo, The Netherlands). During the purification process, an on-column DNase I digestion step with the RNase-Free DNase Set (Qiagen) was incorporated to remove any contaminating genomic DNA. The quantity and quality of the isolated RNA were subsequently evaluated with a NanoDrop 2000 spectrophotometer (Thermo Fisher Scientific Inc., Waltham, MA, USA). For complementary DNA (cDNA) synthesis, 3 µg of RNA served as the template, and reverse transcription was carried out using M-MLV Reverse Transcriptase (Promega Corporation, Madison, WI, USA). The resulting cDNA samples were employed for the analysis of mRNA expression, with gene-specific, intron-spanning primers designed to ensure amplification accuracy. Quantitative real-time PCR (qRT-PCR) was conducted using the RT PCR Mix SYBR reagent (A&A Biotechnology Ltd., Gdańsk, Poland) and performed on a LightCycler 480 platform (Roche Holding AG, Basel, Switzerland). Relative gene expression levels were calculated by the ΔΔCt method, with β-actin used as the internal reference gene for normalization. The ΔΔCt method was applied using Ctrl group samples as the calibrator. The specificity of each amplicon was confirmed by resolving qRT-PCR products on 2.5% agarose gels, followed by visualization with ChemiDoc-XRS+ with Image Lab Software 6.0 (Bio-Rad Laboratories, Hercules, CA, USA). All primer sequences utilized in this study are listed in Table 2. Universal primers were designed for the amplification of the rat and mouse RGN transcripts based on the high sequence conservation of the gene’s open reading frame.

### 4.6. Western Blot Analysis

Podocyte cultures and rat tissue samples were lysed using 1× RIPA buffer (Merck KGaA, Darmstadt, Germany) supplemented with protease inhibitors (Merck KGaA) to prevent protein degradation. Primary and secondary antibodies were obtained from Abclonal (Düsseldorf, Germany), Santa Cruz Biotechnology (Heidelberg, Germany) and Sigma-Aldrich, Merck KGaA. Rat tissues were homogenized on ice at 4 °C using a Dounce homogenizer. After lysing, samples were centrifuged at 18,000 rpm for 20 min at 4 °C to pellet cellular debris. The supernatants were then collected and stored at −80 °C until further analysis. Protein samples were separated on 10% SDS-PAGE gels and transferred to PVDF membranes for immunoblotting. The membranes were blocked in Tris-buffered saline containing 5% non-fat dry milk for 90 min, followed by overnight incubation at 4 °C with primary antibodies (details in Table 3). Detection was carried out with HRP-conjugated secondary antibodies (details in Table 3) and enhanced chemiluminescence reagents (catalog no. #1705061, BioRad, Hercules, CA, USA) by ChemiDoc^TM^ XRS+ Image Lab Software 6.0 (BioRad). Protein band intensities were quantified by densitometry using ImageJ software, version 1.52a, and normalized to β-actin levels. β-actin served as a loading control to normalize protein expression across samples. All results were standardized relative to NG conditions.

### 4.7. Immunofluorescence Staining

For immunofluorescence, podocytes were first fixed with 4% paraformaldehyde in phosphate-buffered saline (PBS) for 20 min at room temperature. Permeabilization of the plasma membrane was achieved by incubating the cells with 0.1% Triton X-100 for 45 s. Subsequently, cells were blocked for 1.5 h in a solution containing 2% fetal bovine serum, 2% bovine serum albumin, and 0.2% fish gelatin dissolved in PBS. Primary antibodies (details in Table 3) were then applied overnight at 4 °C. Detection was carried out with fluorescent dye-conjugated secondary antibodies purchased from Thermo Fisher Scientific (details in Table 3). The cell nuclei were visualized by counterstaining with a mounting medium containing DAPI (catalog no. 50011, ibidi GmbH, Gräfelfing, Germany). Fluorescence images were captured using a Nikon Eclipse Ti confocal microscope equipped (Nikon Instruments Inc., Melville, NY, USA) with NIS-Elements software version 5.11.02 (Nikon Instruments Inc.).

### 4.8. Immunohistochemical Staining

Immunohistochemistry was performed on 6 µm sections of renal tissue from Wistar rats that were intraperitoneally injected with citrate buffer (pH 4.6; Ctrl) or streptozotocin (STZ, 80 mg/kg). Kidney tissue sections were deparaffinized and rehydrated by sequential washing with Histochoice Cleaning Agent (Merck KGaA) and graded ethanol solutions (100%, 95%, 90%, 70%, and 50%). Antigen retrieval was achieved by heating the sections in 10 mM citrate buffer (pH 6.0). Non-specific binding was blocked using 5% bovine serum albumin (BSA) in PBS, followed by overnight incubation with the primary anti-RGN antibody (details in Table 3). On the following day, sections were treated with a secondary antibody and then exposed to Signal Stain DAB Substrate (Cell Signaling Technology, Danvers, MA, USA) for 2 min before immediate rinsing in double-distilled water. Hematoxylin was used for counterstaining, after which the samples were dehydrated through ethanol and cleared with Histochoice Cleaning Agent. Images were captured using a Nikon Eclipse Ti light microscope equipped with NIS-Elements software version 5.11.02 (Nikon Instruments Inc.) and protein staining intensity was quantified with ImageJ software (version 1.52a).

### 4.9. Statistical Analysis

Statistical analyses were performed with GraphPad Prism software (version 8.4.3). The distribution of the data was evaluated using the Shapiro–Wilk test to assess normality. Assumptions for parametric testing were met; comparisons between groups were conducted using an unpaired *t*-test. Data are presented as mean ± SEM, and differences were considered statistically significant when *p* < 0.05.

## Figures and Tables

**Table 1:** Assessment of metabolic parameters in healthy and diabetic Wistar rats. The data are expressed as the mean ± SEM, ^1^** *p* = 0.0025, **** *p* < 0.0001, ^2^** *p* = 0.0018 vs. Ctrl rats.

Parameter	Control Rats (*n* = 6)	STZ Rats (*n* = 6)
Body weight (g)	297.2 ± 12.99	231.7 ± 9.952 ^1^**
Urine volume (mL/24 h)	11.92 ± 2.515	167.0 ± 5.791 ****
Blood glucose concentration (mg/dL)	115.7 ± 11.56	380.2 ± 34.36 ****
Urinary albumin excretion (mg/24 h)	0.1595 ± 0.02720	3.336 ± 0.7514 ^2^**

**Table 2:** Primer sequences utilized in this study for human (h), rat (r) and mouse (m) transcript identification.

Primer Signature	Sequence	Accession No.	Gene ID
F: *RGN* (h)	CGGGATGGGATGGACCC	D31815	9104
R: *RGN* (h)	CCGCATAGGAGTAGGGAGCAA		
F: *Rgn*	CGATTCAATGATGGGAAGGTGGA	X69021 (r) U28937 (m)	25106 (r) 19733 (m)
R: *Rgn*	TACAAGGACCCTTGGTCCG		
F: *ACTB* (h)	ATTGGCAATGAGCGGTTC	X00351	60
R: *ACTB* (h)	GGATGCCACAGGACTCCA		
F: *Actb* (r)	CCACCATGTACCCAGGCATT	V01217	81822
R: *Actb* (r)	GGATAGAGCCACCAATCCACACA		
F: *Actb* (m)	CCACCATGTACCCAGGCATT	X03672	11461
R: *Actb* (m)	GATGGAGCCACCGATCCA		

F, forward primer; R, reverse primer.

**Table 3:** Overview of antibodies used in the experiments.

Antibody	Host	Dilution (Application)	Catalog No.	Supplier
Primary antibodies				
Anti-RGN	Rabbit	1:1000 (WB), 1:200 (IHC)	A3350	ABclonal
Anti-RGN (SMP30)	Mouse	1:40 (IF)	sc-390098	Santa Cruz Biotechnology
Anti-SERCA	Rabbit	1:40 (IF)	sc-30110	Santa Cruz Biotechnology
Anti-βactin	Mouse	1:10,000 (WB)	A5441	Sigma-Aldrich, Merck KGaA
Secondary antibodies				
Anti-mouse HRP	Rabbit	1:3333 (WB)	A9044	Sigma-Aldrich, Merck KGaA
Anti-rabbit HRP	Goat	1:3333 (WB), 1:200 (IHC)	A9169	Sigma-Aldrich, Merck KGaA
Alexa Fluor 488 (anti-mouse)	Goat	1:200 (IF)	A11001	Thermo Fisher Scientific
Alexa Fluor 546 (anti-rabbit)	Goat	1:200 (IF)	A11035	Thermo Fisher Scientific

WB, Western blot; IF, immunofluorescence; IHC, immunohistochemistry.

## Data Availability

The original contributions presented in this study are included in the. article. Further inquiries can be directed to the corresponding author.

## Abbreviations

The following abbreviations are used in this manuscript:

ATPase	Adenosine triphosphatase
Ca^2+^	Calcium ions
Ctrl	Control rats injected with citrate buffer vehicle
DKD	Diabetic Kidney Disease
ER	Endoplasmic reticulum
GFB	Glomerular filtration barrier
HG	High glucose
NADPH oxidase	Nicotinamide adenine dinucleotide phosphate oxidase
NG	Normal glucose
NOX4	Nicotinamide adenine dinucleotide phosphate oxidase 4
RGN	Regucalcin
ROS	Reactive oxygen species
SERCA	Sarco/endoplasmic reticulum Ca^2+^ ATPase
SOCE	Store-Operated Calcium Entry
STZ	Streptozotocin-induced diabetic rats
T2DM	Type 2 Diabetic mellitus
TRP6	Transient receptor potential channel member 6
